# *Enterococcus hirae*‐Mediated ZnO and CuO/ZnO Nanoparticles: Synergistic Antimicrobial Combinations Against MDR Pathogens

**DOI:** 10.1155/ijm/1969553

**Published:** 2026-02-01

**Authors:** Lanya K. Jalal, Laila I. Faqe Salih, Payam B. Hassan

**Affiliations:** ^1^ Department of Medical Laboratory Sciences, College of Sciences, Charmo University, Sulaymaniyah, Iraq, charmouniversity.org; ^2^ Department of Biology, College of Science, University of Sulaimani, Sulaymaniyah, Kurdistan Region, Iraq, univsul.edu.iq

**Keywords:** bimetallic nanoparticles, copper oxide, green synthesis, multidrug resistance, waste water, zinc oxide

## Abstract

The rapid emergence of multidrug‐resistant (MDR) pathogens, particularly in hospital wastewater, poses a serious threat to public health and emphasizes the need for alternative antimicrobial strategies. In this study, *Enterococcus hirae*, an environmentally derived strain, was used for the first time in the extracellular green synthesis of zinc oxide nanoparticles (ZnO NPs) and copper oxide/zinc oxide nanoparticles (CuO/ZnO NPs). The nanoparticles were characterized using standard techniques. Ultraviolet–visible (UV‐Vis) spectra, X‐ray diffraction (XRD), Fourier transform infrared spectroscopy (FTIR), field emission scanning electron microscopy (FE‐SEM), transmission electron microscopy (TEM), and energy‐dispersive X‐ray spectroscopy (EDS) confirmed both nanoparticle formation, size, and morphology. Antimicrobial activity against *Staphylococcus aureus* (ATCC 6538), *Morganella morganii*, *Kerstersia gyiorum*, and *Klebsiella pneumoniae* was evaluated using minimum inhibitory concentration (MIC) and minimum bactericidal concentration (MBC) assays, showing a 62.5% greater efficacy of bimetallic NPs than ZnO alone. The 2,2‐diphenyl‐1‐picrylhydrazyl hydrate (DPPH) assay revealed that E‐CuO/ZnO NPs exhibited superior antioxidant activity with the lowest IC_50_ of 5.528 *μ*g/mL, outperforming E‐ZnO NPs, which is attributed to the synergistic effect between ZnO and CuO NPs. The combination of E‐ZnO and E‐CuO/ZnO nanoparticles with ciprofloxacin (CIP) and ceftazidime (CAZ) was evaluated against MDR isolates. Synergistic interactions were observed particularly against *K. pneumoniae*. This study confirms effective *E. hirae*‐mediated synthesis and the enhanced antibacterial and antioxidant potential of CuO/ZnO NPs, supporting eco‐friendly strategies against MDR infections, with synergistic interactions observed with conventional antibiotics, particularly against *K. pneumoniae*, indicating that the nanoparticles can enhance antibiotic efficacy.

## 1. Introduction

The global rise of multidrug‐resistant (MDR) bacterial pathogens presents a formidable challenge to public health, particularly in clinical environments where nosocomial pathogens thrive. Hospital wastewater is a critical hotspot for the proliferation and horizontal transfer of antimicrobial resistance genes among opportunistic and environmental bacterial populations [1]. Hospital effluents (HEs) are classified as a distinct category due to their highly hazardous and toxic nature. These wastewaters typically contain a complex mixture of antibiotics, disinfectants, and drug metabolites, as well as both susceptible and resistant bacterial strains originating from patients in healthcare [2]. Hospital wastewater can serve as a reservoir for the dissemination of resistant bacteria, such as carbapenemase‐producing *Enterobacterales* (CPE) and their associated resistance genes. Among the CPE are critically important pathogens, according to WHO guidelines, necessitating rapid development of new antibiotics [3]. In addition to harboring antibiotic‐resistant bacteria, hospital wastewater is notable for the high prevalence of MDR coliforms capable of strong biofilm formation, which enhances bacterial survival and resistance by limiting antibiotic penetration and facilitating horizontal gene transfer. Overexpression of efflux pumps and the presence of multiple extended‐spectrum beta‐lactamase (ESBL) genes, often in combination, contribute significantly to resistance phenotypes [2]. Nanotechnology offers promising avenues to counteract MDR pathogens, particularly through the development of metal oxide NPs such as ZnO and CuO NPs [4]. These nanoparticles are known to have strong antibacterial properties through several mechanisms, such as the production of reactive oxygen species (ROS), disruption of bacterial membranes, and release of toxic metal [5, 6]. Recent studies indicate that bimetallic nanoparticles, such as CuO/ZnO composites, may outperform monometallic counterparts by exhibiting synergistic antibacterial [7]. The biological production of nanoparticles utilizing microbial agents offers a sustainable, economical, and environmentally friendly alternative to conventional chemical and physical [8] . Although several bacterial species have been explored for this purpose, to the best of our knowledge, the use of *Enterococcus hirae* as a biosynthetic agent for (ZnO) NPs or (CuO/ZnO) as bimetal nanoparticle production has not been previously reported. This study is aimed at investigating and explore the extracellular green synthesis of ZnO and CuO/ZnO NPs using *E. hirae* isolated from hospital wastewater as a biological agent and to evaluate their characterization, antibacterial, and antioxidant efficacy against MDR pathogens obtained from the same source.

## 2. Materials and Methods

### 2.1. Isolation and Identification

Wastewater samples were aseptically collected from Shar and Anwar Sheikha hospitals in Sulaymaniyah, Iraq, and inoculated in nutrient broth (Scharlau, Spain) at 36°C for 24 h. Following incubation, the samples underwent serial dilution and were subsequently inoculated onto nutrient agar plates using the spreading technique (Scharlau, Spain) to isolate pure single colonies [9]. Selected colonies underwent initial identification through manual methods, including biochemical tests such as oxidase (Bioanalyse strip, Turkey) [10] and microscopic examination with Gram staining (Atom, United Kingdom) [11], and macroscopic assessment of colony characteristics like size, shape, elevation, margin, surface texture, and pigmentation by subculturing on selective and differential [12]. To confirm the MDR nature of the bacterial isolates, antibiotic susceptibility was evaluated using the Kirby–Bauer [13]. The antibiotics tested included amoxicillin–clavulanate (20/10 *μ*g), aztreonam (30 *μ*g), ceftazidime (CAZ) (30 *μ*g), imipenem (10 *μ*g), gentamicin (10 *μ*g), ciprofloxacin (CIP) (10 *μ*g), sulfamethoxazole–trimethoprim (1.25/23.75 *μ*g), and piperacillin/tazobactam (100/10 *μ*g) (HiMedia, India).

### 2.2. Molecular Identification of Bacterial Strains

Genomic DNA was isolated from each bacterial isolate using Presto Mini gDNA Bacteria Kit (Geneaid Biotech Ltd., New Taipei City, Taiwan), according to the manufacturer′s instructions. Using universal bacterial 16S rRNA primers, graphical 7F (5 ^′^‐AGAGTTTGATYMTGGCTCAG‐3 ^′^) and 1510R (5 ^′^‐ACGGYTACCTTGTTACGACTT‐3 ^′^), [14],bacterial isolates were analyzed by classical PCR (polymer chain reaction technique). PCR amplification was performed under the following thermal cycling conditions: an initial denaturation at 95°C for 5 min, followed by 35 cycles consisting of denaturation at 95°C for 30 s, annealing at 60°C for 30 s, and extension at 72°C for 3 min, with a final extension step at 72°C for 5 min. A Gel (Carl ROTH, Germany) and Gel DocTM XR + imaging system (US) was used to run the PCR product [15]. DNA sequencing using a 3500xL Genetic Analyzer (Applied Biosystems) with the same forward and reverse PCR primer strands from Macrogen Inc. (Daejeon, Republic of Korea), the amplicons of the four bacterial samples were sequenced using the Sanger technique. All of the sequences acquired for this investigation were applied to multiple sequence alignment using the BioEdit software Version 7.2.5. After being assigned accession numbers by GenBank (National Center for Biotechnology Information, Bethesda, Maryland, United States), the consensus sequences were analyzed and compared against each other as well as with existing sequences in the GenBank database using the BLAST tool (https://www.ncbi.nlm.nih.gov/Blast.cgi). The MEGA X software program, Version 10.7.1, was used to generate the phylogenetic tree based on the 16S rRNA sequences [16]. The tree was constructed based on the maximum likelihood method [17].

### 2.3. Biological Synthesis of Monometallic Zinc Oxide (E‐ZnO NPs) and Bimetallic Zinc Oxide/Copper Oxide (CuO/ZnO NPs)

**Figure 1:**
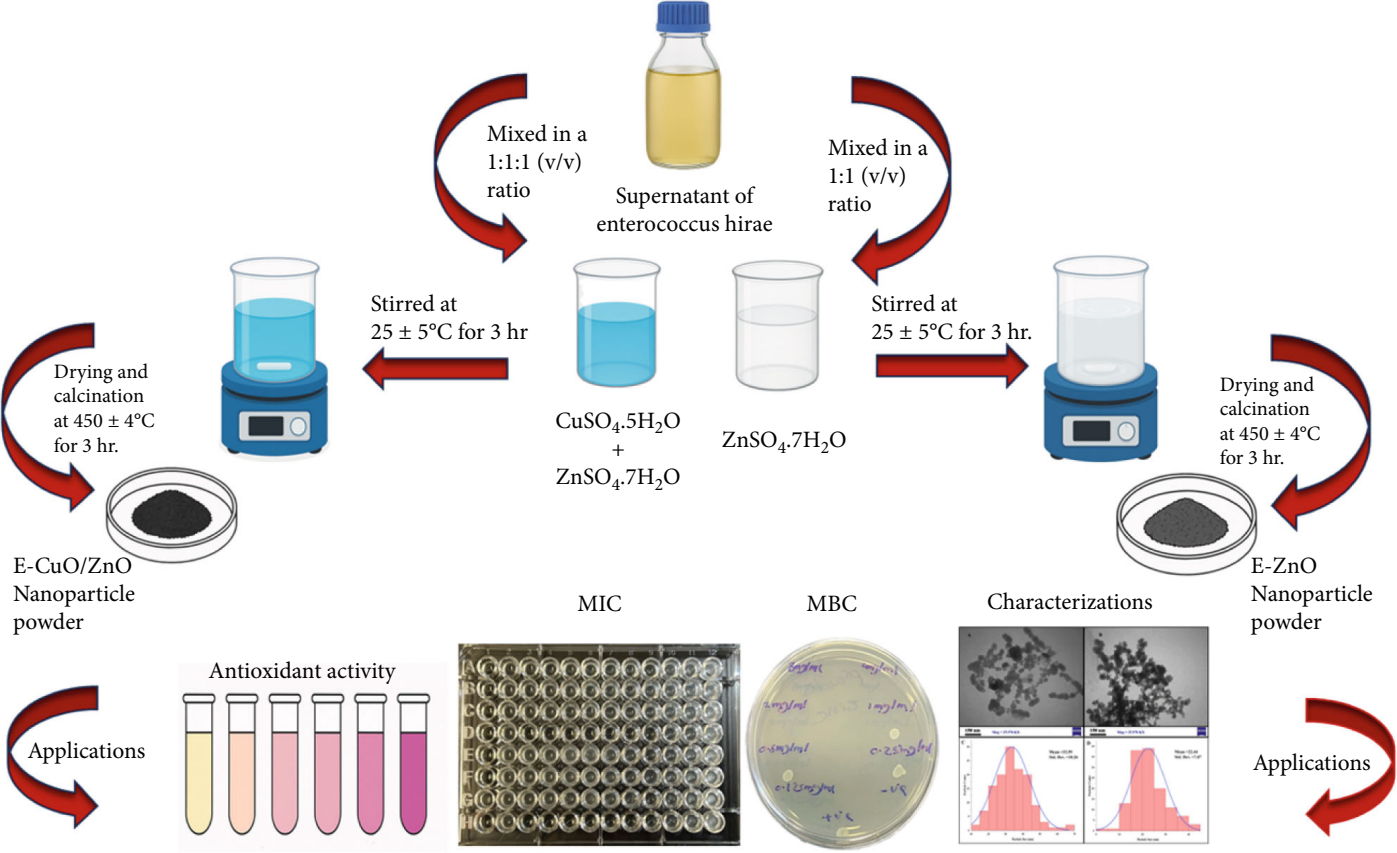
Schematic diagram of the extracellular biosynthesis of *Enterococcus hirae*‐mediated monometallic zinc oxide (E‐ZnO) and *E. hirae*‐mediated copper oxide/zinc oxide (E‐CuO/ZnO) nanoparticles, including their characterizations and applications.

In pilot‐scale screening, one of the bacterial isolates was chosen for biosynthesizing both monometallic and bimetallic nanoparticles. Briefly, a 0.1‐Molar (M) solution of zinc sulfate heptahydrate (ZnSO₄·7H₂O) (Slchem, India) was prepared. In a similar manner, 0.1 M of copper sulfate pentahydrate (CuSO₄·5H₂O) (SDFCL, India) was prepared independently. The two metal salt solutions were subsequently mixed in a 1:1 volumetric ratio to yield equimolar concentrations of both metal ions for the synthesis of bimetallic NPs. Extracellular biosynthesis of *E. hirae*‐mediated monometallic zinc oxide nanoparticles (E‐ZnO NPs) and bimetallic *E. hirae*‐mediated copper oxide/zinc oxide nanoparticles (E‐CuO/ZnO NPs) was accomplished using its cell‐free supernatant. According to [18] with some modifications, 100 mL of nutrient broth was used to freshly inoculate Erlenmeyer flasks with pure colonies of the chosen bacterial isolate. The flasks were incubated for 72 h at 37°C/180 rpm on a shaking incubator. The cell‐free supernatants were obtained by centrifugation at 10,000 rpm for 10 min. For the full synthesis of both metallic nanoparticles, the supernatants were combined with 0.1 M of metal solutions (1:1, v/v), and the pH of the mixtures was then adjusted to eight by adding 1 M sodium hydroxide (NaOH). The mixture was stirred for 3 h at 25°C ± 5°C, after which it was kept in the dark and incubated at 30°C for 96 h. Following drying, the resultant powder was gathered and put through an hour‐long annealing procedure (calcination) in a muffle furnace at 450°C ± 4°C. The powder was washed with sterile distilled water three times (Figure 1) [19].

### 2.4. Characterization of E‐ZnO NPs and E‐CuO/ZnO NPs

The absorption spectrum was recorded using an Ultraviolet–visible (UV‐Vis) spectrophotometer (Agilent Technologies Cary 60, United States) across a wavelength range of 200–1200 nm [20]. The crystalline structure was obtained by X‐ray diffraction (XRD) (PAN Analytical Xpert Pro, Netherlands) using copper *K*‐alpha (Cu‐K*α*) radiation with a nickel filter at 50.0 mA and 40.0 kV [21, 22]. Fourier transform infrared spectroscopy (FTIR) (Thermo Scientific Nicolet iS10 FT‐IR spectrometer) was performed in the 4000–500 cm^−1^ range to identify surface functional groups on biosynthesized NPs [23]. The topology and elemental compositions of NPs were ascertained using field emission scanning electron microscopy (FE‐SEM) (Quanta 4500) with energy‐dispersive X‐ray (EDS) (Bruker, Germany). Nano powders were applied to a stub using carbon tape, fixed, gold‐coated by sputtering, and [24]. In addition, the morphology of monometallic and bimetallic NPs was investigated using transmission electron microscopy (TEM) (JEM‐2100, JEOL, Japan); powdered samples were dispersed in ethanol, placed onto a TEM grid, and dried before analysis [25].

### 2.5. Antibacterial Activity Assay

The biosynthesized monometallic and bimetallic NPs were tested for bacterial activity by minimum inhibitory concentration (MIC) and minimum bactericidal concentration (MBC). The nanoparticles were investigated for effectiveness against three local MDR bacterial strains alongside *Staphylococcus aureus* ATCC (6538). Nanoparticle solutions were sonicated for 10 min, then serially diluted twofold from 8 to 0.125 mg/mL. Following the Clinical and Laboratory Standards Institute (CLSI) [26] and the method described by [27], 100 *μ*L of each dilution was dispensed into a 96‐well microplate (triplicates), with 90 *μ*L of Mueller–Hinton broth (Scharlau, Spain) and 10 *μ*L of a 0.5 McFarland bacterial suspension added to each well. Then, the microplates were incubated at 37°C for 20 h, and the optical density of the bacterial culture was read at 600 nm using a microplate spectrophotometer (BioTek *μ*Quant, United States). Following the MIC analysis, the MBC was determined by spotting 5 *μ*L from all wells onto nutrient agar plates, then the plates were incubated at 37°C for 24 h. The MBC was defined as the lowest concentration exhibiting no visible growth upon [28].

### 2.6. Determination of MIC of Antibiotics and Their Combination With Nanoparticles Against MDR Isolates

According to CLSI guidance with some modifications, the antimicrobial activity of CAZ and CIP, (Sigma‐Aldrich, Germany) was assessed against MDR isolates *(Morganella.morganii, Klebsiella. pneumoniae, and Kersteria.giyorum)* using the broth microdilution method in 96‐well plates. Antibiotic concentrations ranged from CIP 1–32 *μ*g/mL and CAZ 1–64 *μ*g/mL by twofold serial dilutions. E‐ZnO and E‐CuO/ZnO NPs were tested at concentrations from 0.125–8 mg/mL. Antibiotic MIC assays, 20 *μ*L of overnight bacterial suspension (0.5 McFarland) was combined with 20 *μ*L of antibiotics and 160 *μ*L of Mueller–Hinton broth per well. In addition, combination of antibiotics with nanoparticles were conducted, each antibiotic tested individually with both nanoparticle types by mixing 20 *μ*L of bacterial suspension, 20 *μ*L of nanoparticles, 20 *μ*L of antibiotic, and 160 *μ*L of Mueller–Hinton broth per well. The final step for both MIC and combination assays was incubation at 37°C for 20 h, followed by reading the optical density at 600 nm (OD_600_). MICs were defined as the lowest concentration without visible growth. Finally, the fractional inhibitory concentration (FIC) index was calculated [29] to evaluate the interaction between antibiotics and nanoparticles using the following formula:

FIC index FICI=MIC of antibiotic A in combination÷MIC of antibiotic A alone+MIC of nanoparticle A in combination÷MIC of nanoparticle A alone

The FIC index was interpreted as follows: ≤ 0.5 indicates synergism, > 0.5–1 indicates additive effect, > 1–4 indicates indifference, and > 4 indicates antagonism.

### 2.7. Antioxidant Activity

2,2‐Diphenyl‐1‐picrylhydrazyl (DPPH) was prepared as a 0.1‐mM solution in methanol. A total of 100 *μ*L of this was added to 300 *μ*L of nanoparticle dispersions at different concentrations (50, 100, 200, 400, and 800 *μ*g/mL). Mixtures were shaken well and were incubated for 30 min at room temperature. The absorbance was recorded by a UV‐Vis spectrophotometer at 517 nm with ascorbic acid (AA) as the standard. Every experiment was repeated three times to ensure accuracy [30]. The percentage of DPPH radical scavenging was calculated using the following equation:

Scavenging activity %=100×Ac−As÷Ac

where (Ac) is the absorbance of the control and (As) is the absorbance of the sample at 517 nm.

### 2.8. Statistical Methods

The study was performed using a completely randomized design. Data were analyzed through variance analysis using the general linear model (GLM) in SPSS Version 22. Graphical representations were created using OriginLab Version 10.25.212 and GraphPad Prism Version 10.5.774 software. When significant differences among means were detected at the 0.05 level, Duncan′s multiple range test (1955) was applied for comparison. A *p* value of less than 0.05 was regarded as statistically significant.

## 3. Results and Discussion

### 3.1. Isolation and Identification of Bacterial Strains

**Figure 2:**
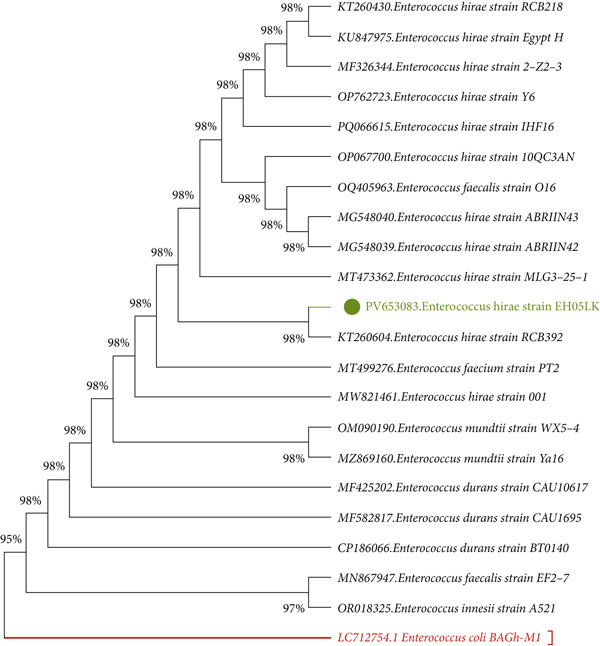
Phylogenetic tree of *Enterococcus hirae* strain (EH05LK) based on partial 16S rRNA gene sequencing.

*E. hirae* was isolated from hospital wastewater in Sulaymaniyah, Iraq. The isolated bacteria were identified using biochemical tests and confirmed by 16S rRNA gene sequencing as *E. hirae* EH05LK with the Accession Number (PV653083.1); sequences were submitted to GenBank with 100% similarity to reference strains in the National Center for Biotechnology Information (NCBI) database. Phylogenetic analysis using the neighbor‐joining method (Figure 2) showed that strain EH05LK is closely related to *E. hirae* RCB392 (KT260604), confirming its taxonomic placement. *E. hirae* is a Gram‐positive facultative anaerobe commonly found in water, soil, and the human gut [31] MDR strains of *K. pneumoniae* (PV653082.1), *K. gyiorum* (PV653081.1), and *M. morganii* (PV653080.1) were also isolated from hospital wastewater and confirmed by molecular sequencing. These clinically relevant pathogens are known for antibiotic resistance and association with healthcare‐related infection studies [32–35]. MDR is characterized by acquired nonsusceptibility to at least one agent across three or more antimicrobial [36]. The antimicrobial susceptibility test was performed using the Kirby–Bauer disk diffusion method [13]. All three MDR strains demonstrated resistance to more than four distinct classes of antibiotics, indicating a high level of multidrug resistance, as detailed in File S1: Table S1 Antibiotic susceptibility test (AST) results of selected bacterial strains by disk diffusion method.

### 3.2. Nanoparticle Synthesis

Extracellular synthesis is preferred over intracellular methods due to higher yield, simplified processing, enhanced separation, and better applicability for industrial applications. *E. hirae*, a lactic acid bacterium (LAB), may facilitate the creation of metal nanoparticles due to its Gram‐positive cell wall composed of peptidoglycan, teichoic acid, lipoteichoic acid, proteins, and polysaccharides, which promote the biosorption and bioreduction of metal ions. Nitrate reductase and nicotinamide adenine dinucleotide (hydrogen) (NADH)‐dependent reductase enzymes reduce Zn^2+^/Cu^2+^ to Zn^0^/Cu^0^ during the extracellular production of ZnO/CuO nanoparticles. Microbial extracellular proteins function as capping agents, wherein amino acids combine with Zn^2+^/Cu^2+^ to form nanoparticles [37–39]. Our findings demonstrate that the bacterial metabolites of *E. hirae*, a Gram‐positive, oxidase‐negative, catalase‐negative bacterium (File S2: Table S1 Biochemical characteristics, Gram reaction, oxidase, and catalase results of *E. hiare*, *Morganella morganii*, *Kersteria gyiorum,* and *Klebsiella pneumoniae*) are capable of effectively reducing zinc sulfate (ZnSO_4_) to zinc oxide (ZnO) NPs and copper sulfate (CuSO_4_) to copper oxide (CuO) NPs. This reduction process was evidenced by a color change of the ZnSO_4_ solution to white, consistent with observations reported by [40], and the formation of a blue, paste‐like structure in the bimetallic solution, as reported by [19]. These visual changes are indicative of successful nanoparticle synthesis and are attributed to surface plasmon resonance (SPR), as described by [41]. Researchers are increasingly focused on the development and fabrication of metal oxide nanoparticles, including CuO and ZnO NPs, due to their promising broad‐spectrum antibacterial properties. Although earlier studies have focused on other bacterial strains, such as *Pseudomonas* spp., for biosynthesis of metal oxide nanoparticles [42], the use of *E. hirae* for the extracellular synthesis of both monometallic ZnO and bimetallic CuO/ZnO NPs has not been reported.

### 3.3. Nanoparticle Characterizations

#### 3.3.1. Ultraviolet–Visible Spectroscopy

Ultraviolet–Visible (UV‐Vis) spectroscopy was utilized to investigate the formation, stability, and optical characteristics of metal nanoparticles. The observed SPR bands at 378 nm for E‐ZnO NPs and at 502 nm, 368 nm for E‐CuO/ZnO NPs confirm the successful synthesis of both E‐ZnO NPs and the bimetallic E‐CuO/ZnO NPs (Figure 3a). These results suggest that incident UV radiation promotes the excitation of electrons from the valence band to the conduction band of the as also mentioned in previous studies [19, 43].

Figure 3(a) Ultraviolet–visible (UV‐Vis) spectroscopy, (b) Fourier transform infrared spectroscopy (FTIR), and (c) X‐ray diffraction (XRD) analyses of E‐ZnO and E‐CuO/E‐ZnO NPs.

**Figure:**
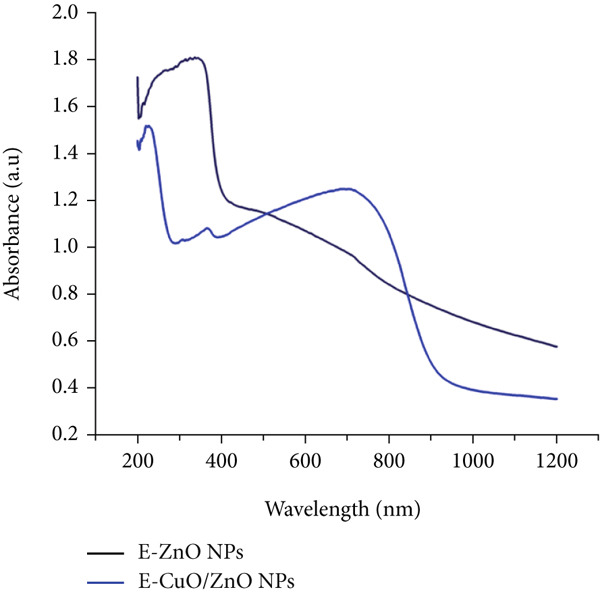
(a)

**Figure:**
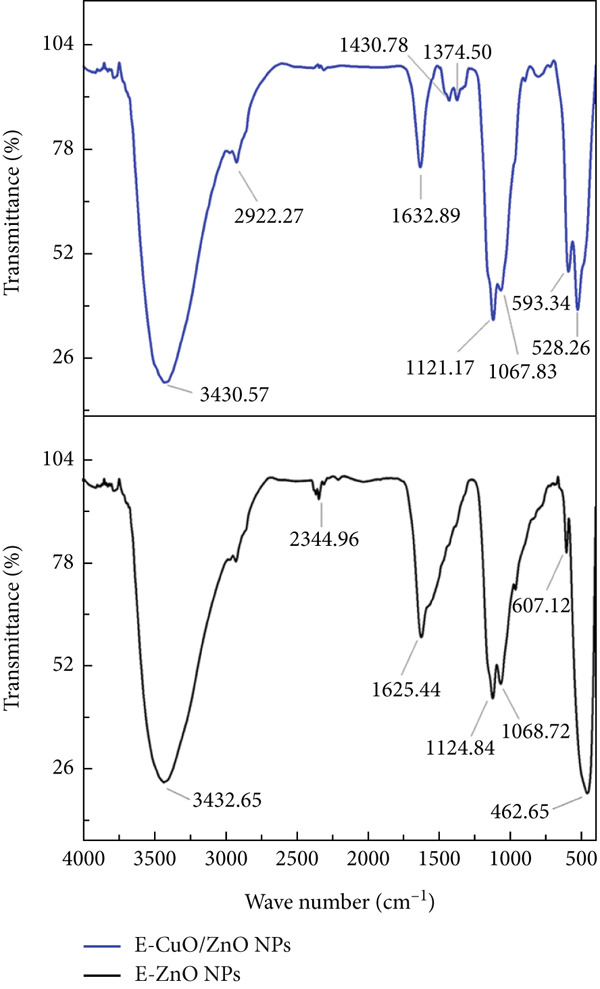
(b)

**Figure:**
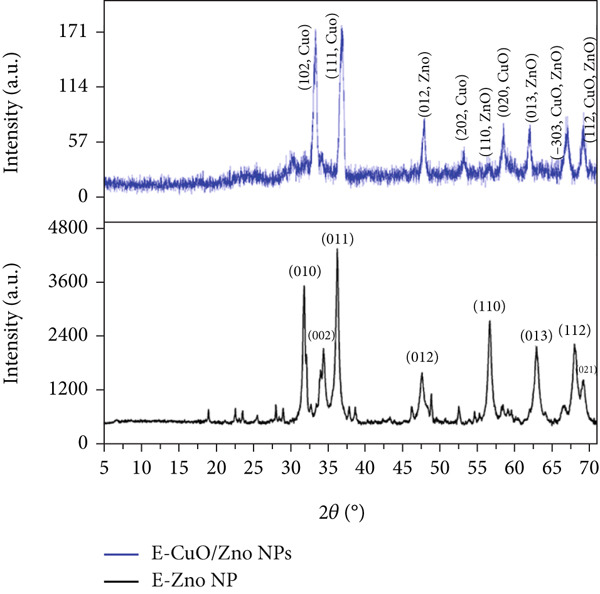
(c)

#### 3.3.2. X‐Ray Diffraction

The XRD pattern of E‐ZnO NPs displayed the characteristic reflections of hexagonal wurtzite ZnO, consistent with the standard reference pattern (ICDD 98‐011‐5755) [44]. In the bimetallic (E‐ZnO/CuO) NPs, additional peaks attributable to CuO (ICDD 98‐065‐3723) also appeared, suggesting the presence of a mixed‐phase structure comprising both ZnO and monoclinic CuO as shown in Figure 3c and File S3: Table S1 XRD results summary. The slight peak shifts observed in the XRD pattern of the E‐CuO/ZnO sample suggest lattice distortion due to interaction between CuO and ZnO, indicating the formation of a nanocomposite [45, 46]. Also, coexistence (overlapping) of these peaks indicates the successful formation of a mixed‐phase bimetallic nanostructure. This pattern confirms the crystalline nature of the synthesized ZnO/CuO NPs [47]. Similar findings have been reported in previous studies involving green synthesis of metal oxide nanoparticles [21, 48].

#### 3.3.3. Fourier Transform Infrared Spectroscopy

FTIR of the biosynthesized E‐ZnO nanoparticles (ZnO NPs) exhibited bands attributable to hydroxyl (O–H) groups, atmospheric carbon dioxide (CO_2_), carbon–carbon or hydroxyl (C=C/–OH) vibrations, carbon–oxygen (C–O) stretching, and zinc–oxygen (Zn–O) bonds, confirming the formation of ZnO (Figure 3b). The bimetallic E‐ZnO/CuO nanoparticles (ZnO/CuO NPs) displayed these ZnO‐related vibrations along with additional features corresponding to carbon–hydrogen (C–H) stretching, carboxylate (COO^−^) groups, carbonyl or amine‐related (C=O/N–H) bending, and metal–oxygen vibrations assignable to both Zn–O and copper–oxygen (Cu–O), indicating successful incorporation of copper (II) oxide (CuO) into the nanocomposite structure as shown in Figure 3b and File S3: Table S2 FTIR results summary. Biomolecules such as phenolics and polyphenols played a critical role in the biosynthesis and stabilization of the E‐ZnO NPs. A broad O–H stretching band indicates the presence of hydroxyl‐rich compounds, likely acting as reducing agents, which is consistent with findings in ZnO NPs synthesized using *P. macrosolen* leaf extract. Similar broad bands around 3450 cm^−1^ have been attributed to phenolic OH groups in green‐synthesized ZnO NPs [49]. A minor absorption band, (CO_2_) observed in the spectrum, is commonly regarded as a background signal in FTIR analysis and is not related to any functional groups present in the ZnO nanoparticles [50]. Additional bands indicated the presence of carbon–carbon and hydroxyl bonds, possibly from polyphenols, as well as C–O stretching vibrations linked to alcohols, ethers, or other oxygen (O)‐containing biomolecules. Notably, characteristic bands for Zn–O confirmed the successful formation of E‐ZnO NPs. These results imply that biomolecules from the synthesis medium participated both in reducing zinc (Zn) ions and in capping the nanoparticles to enhance their stability. Similar findings have been reported in a previous study [51]. The FTIR results confirm that bimetallic E‐CuO/ZnO NPs were successfully synthesized and stabilized by biomolecules from the bacterial extract. The presence of aliphatic chains, carboxylate groups, and amide or amine‐related vibrations suggests that proteins, fatty acids, and other organic compounds played a key role in the nanoparticle formation. Metal–oxygen bond signatures indicate the formation of both Zn–O and Cu–O, verifying the bimetallic structure. These findings highlight the dual role of bacterial metabolites in reducing metal ions and capping the nanoparticles for stability, as reported in previous studies [22, 46, 52, 53] .

#### 3.3.4. FE‐SEM

FE‐SEM (Figure 4c) revealed E‐ZnO NPs as predominantly spherical with uniform size and minimal agglomeration, whereas E‐CuO/ZnO NPs (Figure 4a) exhibited varied morphologies and aggregation, suggesting CuO incorporation affects nucleation and growth [54]. Also, slight aggregation appeared and it is typical in biologically synthesized metal oxides [55]

Figure 4(a) and (c), field emission scanning electron microscopy (FE‐SEM) images (scale bar = 200 nm), of E‐CuO/ZnO and E‐ ZnO NPs, respectively, along with their EDS spectra in (b) and (d).

**Figure:**
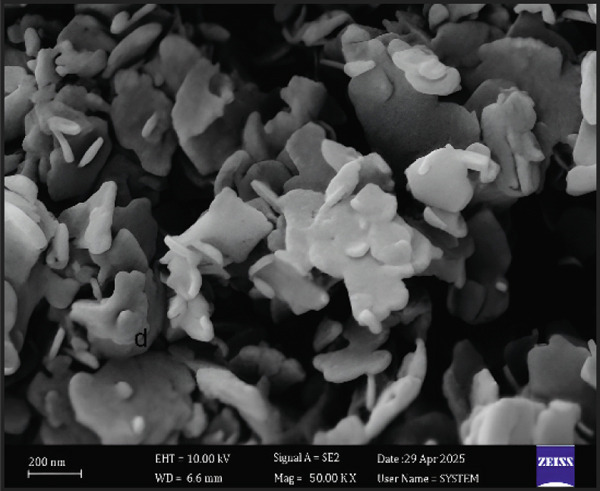
(a)

**Figure:**
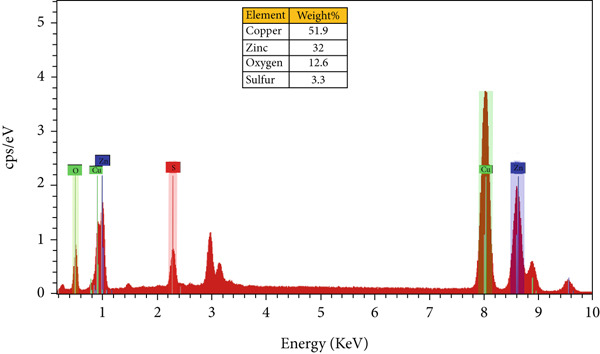
(b)

**Figure:**
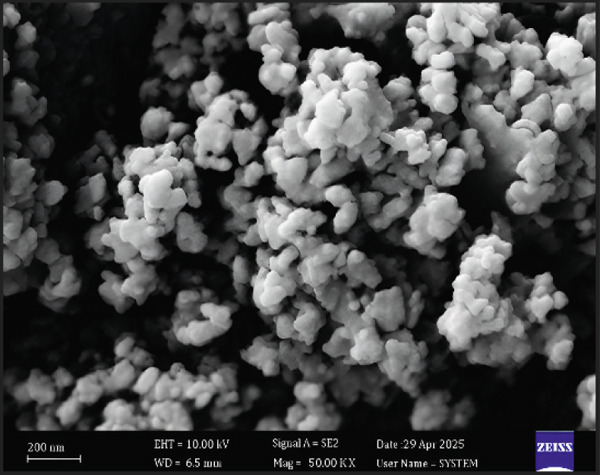
(c)

**Figure:**
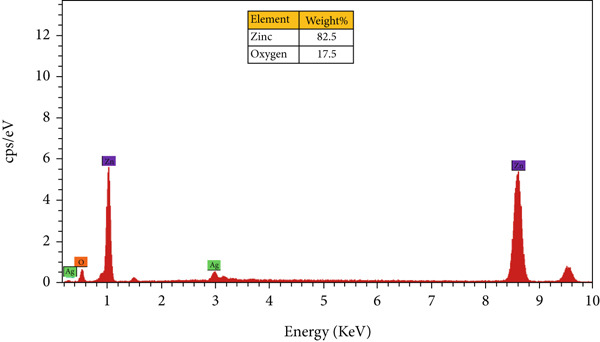
(d)

#### 3.3.5. EDS

EDS spectra were employed to confirm the elemental composition and distribution in both monometallic and bimetallic E‐ZnO/CuO NPs. The EDS spectrum of E‐ZnO nanoparticles showed the strongest peak at (1 keV), confirming the presence of Zn as the dominant element (82.5 weight %) with minor contributions from O (17.5%) due to surface oxide formation. Minor silver (Ag) signals originated from the FESEM coating as illustrated in Figure 4d and File S3 Table S3 EDS results summary. [56]. The bimetallic E‐ZnO/CuO NPs showed characteristic Zn and copper (Cu) peaks, along with O (Figure 4b). The sulfur peak originates from the metal salts used during synthesis. In the bimetallic, Cu (51.9 weight percentage) and Zn (32 weight percentage) are the dominant elements, confirming the successful incorporation of both metals. Similar kinds of results have been reported in previous studies for the biosynthesis of monometallic and bimetallic nanoparticles [57, 58].

#### 3.3.6. TEM

TEM analysis revealed that E‐ZnO NPs (Figure 5a) predominantly exhibited spherical to pseudospherical shapes with uniform surface morphology. In contrast, the TEM image of bimetallic E‐CuO/ZnO NPs (Figure 5b) was smaller in size (mean 22.44 nm, standard deviation [Std.Dev] 7.47) (Figure 5d) compared with E‐ZnO NPs (mean 33.59 nm, SD 10.27) (Figure 5c), displayed slightly irregular shapes, and tended to form compact clusters with some degree of agglomeration. The narrower particle size distribution observed in the bimetallic nanoparticles is likely due to modified nucleation and growth dynamics caused by the simultaneous presence of two metal precursors [59]. This refined size and morphology are advantageous for enhancing surface‐related properties, such as antibacterial activity [60]. These morphological characteristics align with findings reported by [58, 61].

Figure 5Transmission electron microscopy (TEM) images and corresponding particle size distribution histograms of E‐ZnO and E‐CuO/ZnO NPs with their mean and standard deviation (Std.Dev) in (a,b) and (c,d), respectively.

**Figure:**
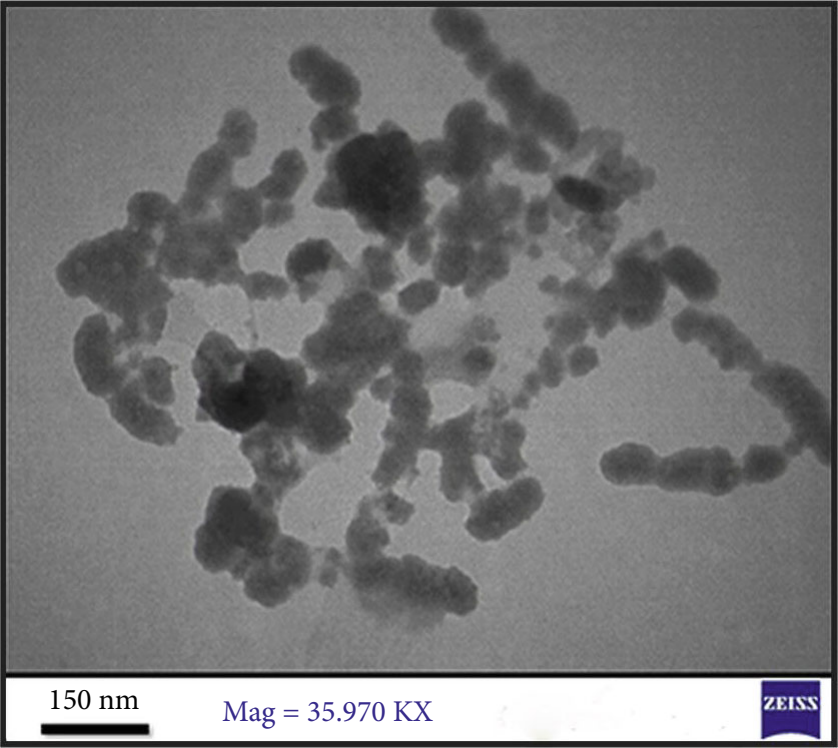
(a)

**Figure:**
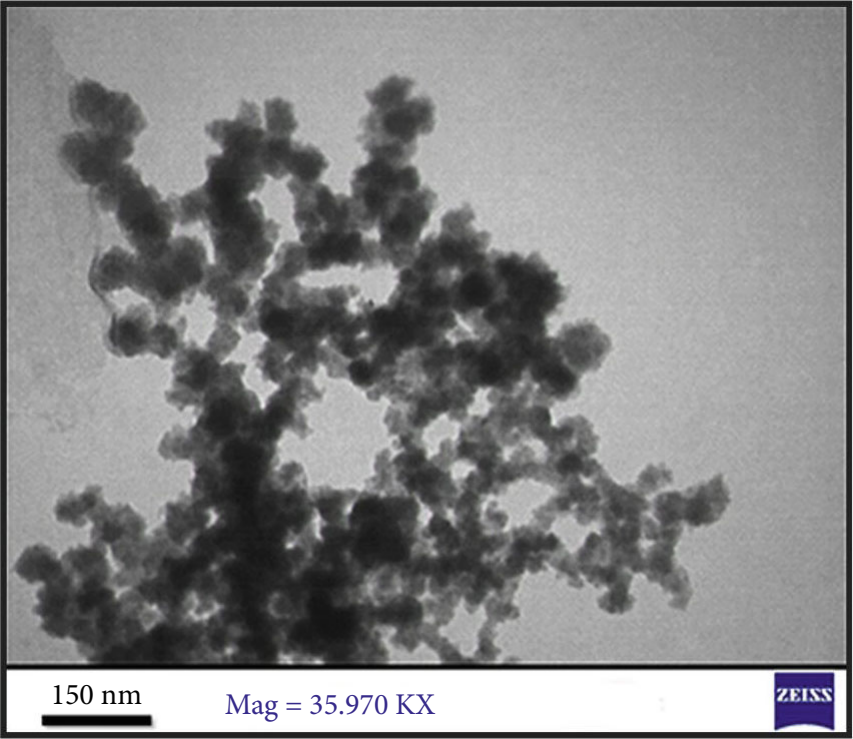
(b)

**Figure:**
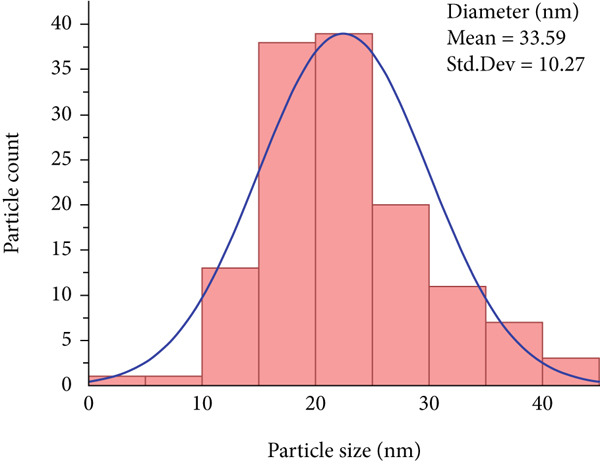
(c)

**Figure:**
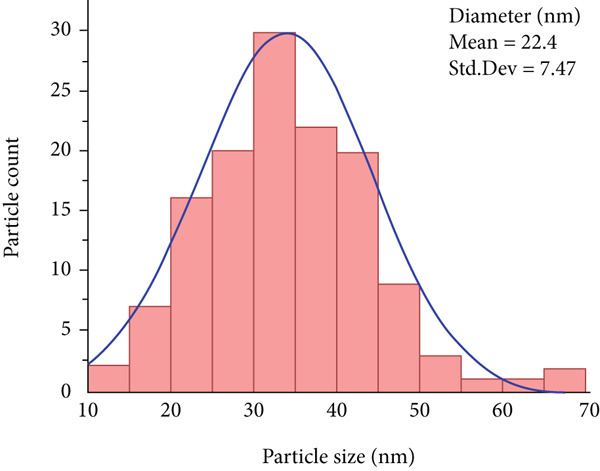
(d)

### 3.4. Antibacterial Activity

**Figure 6:**
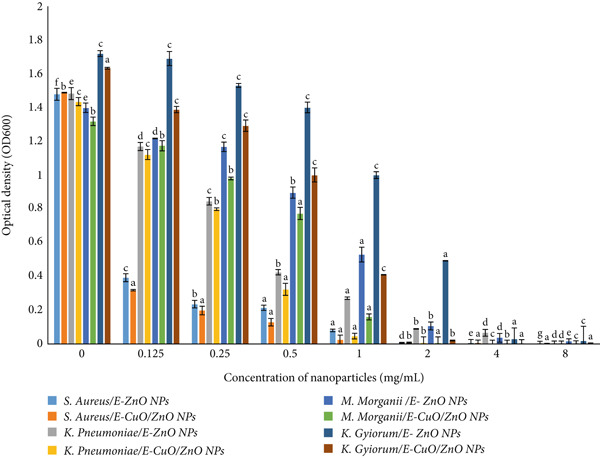
The antibacterial activity of E‐ZnO and E‐CuO/ZnO NPs against MDR isolates (*Morganella morganii*, *Klebsiella pneumoniae*, and *Kersteria gyiorum)* and *Staphylococcus aureus* ATCC (6538) was evaluated by determining the minimum inhibitory concentration (MIC) of the nanoparticles in mg/mL (OD: optical density). Lowercase letters represent statistical groupings determined using one‐way ANOVA followed by Duncan multiple range test. Columns that share the same letter are not significantly different, whereas columns with different letters indicate significant differences.

**Figure 7:**
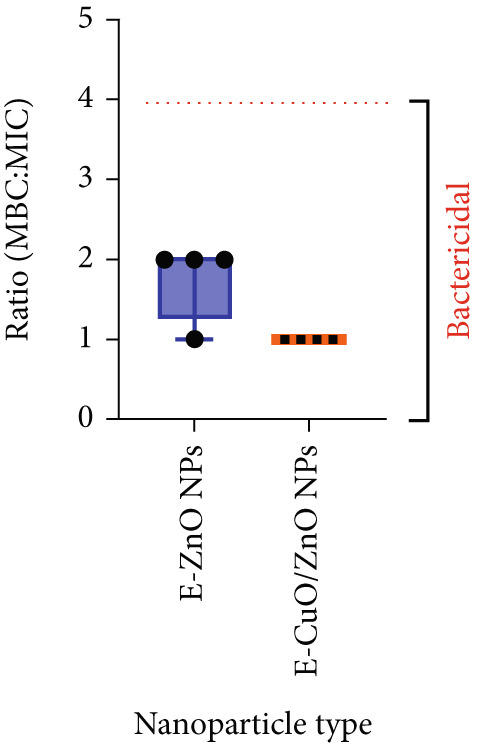
Minimum bactericidal concentration/minimum inhibitory concentration (MBC/MIC) ratios of E‐ZnO and E‐CuO/ZnO against the three MDR isolates and *Staphylococcus aureus* ATCC (6538). MBC/MIC in the range of (0–4) is considered as bactericidal.

In this study, both monometallic and bimetallic nanoparticles synthesized via a biological method were investigated against locally isolated MDR bacterial pathogens from hospital wastewater in Sulaymaniyah, Iraq. The standardized broth microdilution method was employed to assess antimicrobial susceptibility by determining the MIC and MBC [62]. The MIC represents the lowest concentration at which visible bacterial growth is inhibited, whereas the MBC corresponds to the minimum concentration that eliminates ≥ 99.9% of the bacteria [19]. The antibacterial activities of monometallic E‐ZnO NPs and bimetallic E‐CuO/ZnO NPs were tested against *M. morganii, K. gyiorum, K. pneumoniae*, and *S. aureus* ATCC (6538) strain (Figure 6). Bimetallic E‐CuO/ZnO NPs exhibited MIC values of 1 mg/mL for *S. aureu*s and 2 mg/mL for all MDR strains, with identical MBC values, indicating strong bactericidal activity. Compared with the bimetallic NPs, the monometallic E‐ZnO NPs required higher concentrations to inhibit and kill the bacteria. Their MICs were 2 mg/mL for *S. aureus* ATCC (6538), 4 mg/mL for *M. morganii* and *K. gyiorum*, and 8 mg/mL for *K. pneumoniae*. The MBCs were 4 mg/mL for *S. aureus* ATCC 6538 and 8 mg/mL for all MDR strains. All MBC/MIC ratios observed in this study were ≤ 4, indicating bactericidal effects for both types of nanoparticles tested as shown in (Figure 7). According to widely accepted standards, including CLSI [26] and other established studies [63], an MBC/MIC ratio of 4 or less defines bactericidal activity. This definition means the MBC does not exceed four times the MIC. Therefore, the nanoparticles effectively killed the target bacterial pathogens. On average, the bimetallic NPs showed 62.5% greater antibacterial efficacy compared with the monometallic formulation. Lowercase letters above the columns indicate significant differences at *p* < 0.05.Our findings confirm the presence of antibacterial activity in both nanoparticle types, with bimetallic E‐CuO/ZnO NPs demonstrating superior efficacy. This is consistent with previous studies reporting enhanced antimicrobial performance of bimetallic formulations due to synergistic effects between metals [7, 64]. The improved activity may be attributed to synergistic interactions, increased ROS generation, and ion synergy [54]. Antibacterial efficacy is strongly influenced by nanoparticle size, morphology, surface charge, and internal composition [65]. Zn and Cu ions are known to have several antibacterial mechanisms, including disrupting bacterial metabolism and generating ROS, leading to growth inhibition and cell death [66].

### 3.5. Fractional Inhibitory Concentration Index (FICI) of E‐ZnO And E‐CuO/ZnO NPs in Combination With CIP and CAZ

**Figure 8:**
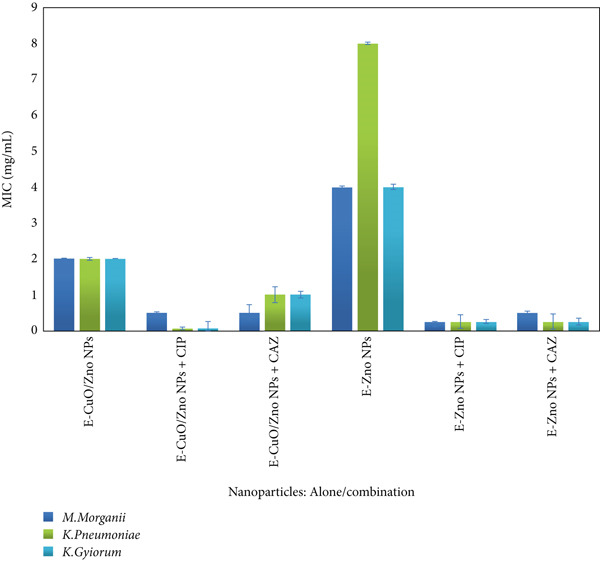
Minimum inhibitory concentration (MIC—mg/mL) of E‐ZnO and E‐CuO/ZnO NPs alone and in combination with antibiotics (CIP and CAZ) against *Morganella morganii*, *Klebsiella pneumoniae,* and *Kersteria gyiorum* bacterial strains.

In the present study, the antimicrobial interactions of E‐ZnO and E‐CuO/ZnO nanoparticles with CIP and CAZ were evaluated using the checkerboard assay, and the FICI was calculated against MDR isolates of *M. morganii*, *K. pneumoniae*, and *K. gyiorum.* The MIC values of CIP alone were 2, 8, and 4 *μ*g/mL for *M. morganii*, *K. pneumoniae*, and *K. gyiorum,* respectively, whereas the MIC values of CAZ were 16, 32, and 8 *μ*g/mL for the same isolates. For nanoparticles alone as mentioned in Section 3.4, E‐CuO/ZnO NPs exhibited MIC values of 2 mg/mL across all three isolates, whereas E‐ZnO NPs showed MICs of 4, 8, and 4 mg/mL for *M. morganii*, *K. pneumoniae*, and *K. gyiorum,* respectively. The FIC analysis showed that both E‐ZnO and E‐CuO/ZnO nanoparticles exhibited the strongest synergistic effects when combined with either CIP or CAZ against K. pneumoniae. In contrast, interactions with *M. morganii* and *K. gyiorum* were mostly additive or indifferent, as shown in Table. 1. Also, a previous study reported that ZnO NPs enhanced antibiotic activity through synergistic interactions using the checkerboard assay [67]. Overall, the MIC values of nanoparticles were reduced when tested in combination compared with their individual MICs (Figure 8), indicating that the nanoparticles enhanced antibiotic efficacy, particularly against *K. pneumoniae* where multiple synergistic interactions were recorded. The enhanced activity may result from targeting distinct biological pathways, from inhibiting different steps within the same pathway, or from acting on the same target through complementary mechanisms that increase overall efficacy [68]. Antibiotic combination therapy involves the concurrent use of multiple agents to manage infections. Such combinations can be more effective in overcoming bacterial resistance compared with individual drugs. The reduction in MIC values when nanoparticles were combined with antibiotics highlights a clinically relevant advantage; lower doses of nanoparticles are required to achieve the same or greater antibacterial effect. This is especially important in minimizing potential nanoparticle‐related cytotoxicity, while at the same time restoring the therapeutic efficacy of antibiotics that are otherwise less effective against MDR pathogens [69, 70]. These results underscore the potential of nanoparticle/antibiotic combinations as a valuable therapeutic approach for combating high‐risk MDR pathogens.

**Table 1:** Fractional inhibitory concentration index (FICI) values showing the interactions between nanoparticles (E‐CuO/ZnO NPs or E‐ZnO NPs) and antibiotics (CIP, CAZ) against MDR bacterial strains. Interpretation: synergistic (FICI ≤ 0.5), additive (0.5 < FICI ≤ 1), indifferent (1 < FICI ≤ 4).

**Antimicrobial agents**	***M. morganii***	***K. pnemoniae***	***K. gyiorum***
E/CuO/ZnO NPs + CIP	1.5 (Indifferent)	0.28 (Synergistic)	0.5 (Synergistic)
E‐CuO/ZnO NPs + CAZ	0.37 (Synergistic)	0.75 (Additive)	1 (Additive)
E‐ZnO NPs + CIP	1 (Additive)	0.5 (Synergistic)	0.56 (Additive)
E‐ZnO NPs + CAZ	0.25 (Synergistic)	0.28 (Synergistic)	0.56 (Additive)

### 3.6. Antioxidant Activity

**Figure 9:**
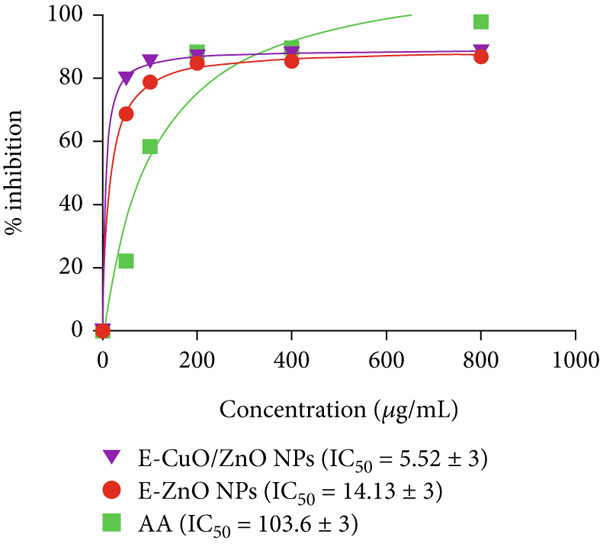
Dose‐response curve for the antioxidant activity of AA (ascorbic acid), E‐ZnO, and E‐CuO/ZnO NPs using the DPPH assay with their respective half maximal inhibitory concentration (IC_50_) values.

DPPH assay was performed to assess the antioxidant potential of the synthesized nanoparticles. The antioxidant activity of all samples exhibited a clear increase with concentration, as shown in Figure 9. The antioxidant potential of E‐ZnO and E‐ZnO/CuO NPs, compared with AA, a standard antioxidant. At lower concentrations (50 and 100 *μ*g/mL), both monometallic and bimetallic NPs showed higher antioxidant activity than AA. The half maximal inhibitory concentration (IC_50_) of AA was (103.6 ± 3) *μ*g/mL, and at 800 *μ*g/mL, the inhibition rate was 97.89%. The bimetallic E‐CuO/ZnO NPs exhibited the lowest IC_50_ value of (5.528 ± 3) *μ*g/mL, demonstrating superior antioxidant activity. At the highest concentration (800 *μ*g/mL), E‐CuO/ZnO NPs showed 88.31% inhibition. In contrast, E‐ZnO NPs displayed a higher IC_50_ of (14.13 ± 3) *μ*g/mL, with 86.89% inhibition at 800 *μ*g/mL, indicating moderate antioxidant activity. These results suggest a synergistic effect between Zn and Cu that improves radical neutralization, as also reported [46]. This synergistic effect may be due to the transfer of free charges between the CuO and ZnO NPs to the DPPH radicals. Furthermore, the improved antioxidant activity of the synthesized E‐CuO/ZnO NPs could be attributed to its higher surface area to volume ratio and smaller particle size [71], as observed in the TEM analysis (Figure 5). Although many studies have reported the synthesis and applications of the CuO/ZnO nanocomposite, only a limited number have investigated its antioxidant properties. Consequently, the biosynthesized E‐CuO/ZnO NPs shows potential as a promising antioxidant treatment for diseases associated with oxidative stress.

## 4. Conclusion

This study is the first to report the successful application of *E.hirae* for the extracellular biosynthesis of both monometallic ZnO and bimetallic CuO/ZnO NPs under ambient conditions. The synthesized nanoparticles displayed distinct morphologies, high crystallinity, and effective bactericidal activity, particularly against MDR strains isolated from hospital wastewater. Notably, the bimetallic E‐CuO/ZnO NPs exhibited superior antibacterial efficacy, highlighting the synergistic advantage of combining metal oxides. In addition, their antioxidant potential surpassed that of the standard control, demonstrating a remarkable radical scavenging capacity. Synergistic effects were also observed when nanoparticles were combined with conventional antibiotics, significantly enhancing antibacterial performance against resistant pathogens. These findings underscore the dual antimicrobial and antioxidant promise of biogenic E‐CuO/ZnO NPs, emphasizing their potential as eco‐friendly, multifunctional nanomaterials for managing MDR pathogens and oxidative stress‐related challenges. Future work will focus on optimizing synthesis parameters, evaluating cytotoxicity for biomedical applications, and exploring broader‐spectrum therapeutic and environmental uses of these biogenic NPs.

## Ethics Statement

The authors have nothing to report.

## Conflicts of Interest

The authors declare no conflicts of interest.

## Author Contributions

Lanya K. Jalal: investigation (practical work), statistical analysis, data curation, and writing of the original draft. Laila I. Faqe Salih and Payam B. Hassan: conceptualization, supervision, project guidance, reviewing, and editing.

## Funding

No funding was received for this manuscript.

## Supporting Information

Additional supporting information can be found online in the Supporting Information section.

## Supporting information


**Supporting Information 1** File S1 includes antibiotic susceptibility test (AST) results of selected bacterial strains by disk diffusion method.

**Supporting Information 2** File S2 shows biochemical characteristics (Gram reaction, oxidase, and catalase results) of selected bacterial strains.

**Supporting Information 3** File S3 contains physicochemical characterization of synthesized nanoparticles, including Table S1: X‐ray diffraction (XRD) results summary, Table S2: Fourier transform infrared spectroscopy (FTIR) results summary, and Table S3: Energy‐dispersive X‐ray spectroscopy (EDS) results summary.

## Data Availability

All data generated or analyzed during this study are included in this published article and its supporting information files. Additional details can be obtained from the corresponding authors upon reasonable request.
