# Supplementary material for: Enterococcus hirae‐Mediated ZnO and CuO/ZnO Nanoparticles: Synergistic Antimicrobial Combinations Against MDR Pathogens
Source: Int J Microbiol. 2026 Feb 1;2026:1969553. doi: 10.1155/ijm/1969553 (PMC12862103; doi:10.1155/ijm/1969553)
Supplement: Supplementary file 1 — Supporting Information 1 File S1 includes antibiotic susceptibility test (AST) results of selected bacterial strains by disk diffusion method. [file IJM-2026-1969553-s002.docx]

**Supplementary File (S1)**

***Enterococcus hirae*-Mediated ZnO and CuO/ZnO Nanoparticles: Synergistic Antimicrobial Combinations Against MDR Pathogens.**

Lanya K. Jalal ^1^, Laila I. Faqe Salih^1^, Payam B. Hassan^2*^

^1^Department of Medical Laboratory Sciences, College of Sciences, Charmo University, Sulaymaniyah 46001, Iraq

^2^Department of Biology, College of Science, University of Sulaimani, Sulaymaniyah, 46001, Kurdistan Region, Iraq

Author for correspondence: Laila Ibrahim Faqe Salih

Email: Laila.Ibrahim@Chu.edu.iq

**Table 1: Antibiotic Susceptibility Test (AST) Results of Selected Bacterial strains by Disk Diffusion Method.**

| Bacterial Species |  | AMC | CIP | CAZ | CN | IPM | SXT | AT | PIT |
| --- | --- | --- | --- | --- | --- | --- | --- | --- | --- |
| *K.pneumoniae* |  | R | R | R | S | R | R | R | R |
| *M.morganii* |  | R | R | R | S | R | S | R | R |
| *K.gyiorum* |  | R | R | R | S | S | R | S | R |
