# Supplementary material for: Enterococcus hirae‐Mediated ZnO and CuO/ZnO Nanoparticles: Synergistic Antimicrobial Combinations Against MDR Pathogens
Source: Int J Microbiol. 2026 Feb 1;2026:1969553. doi: 10.1155/ijm/1969553 (PMC12862103; doi:10.1155/ijm/1969553)
Supplement: Supplementary file 2 — Supporting Information 2 File S2 shows biochemical characteristics (Gram reaction, oxidase, and catalase results) of selected bacterial strains. [file IJM-2026-1969553-s001.docx]

**Supplementary File (S2)**

***Enterococcus hirae*-Mediated ZnO and CuO/ZnO Nanoparticles: Synergistic Antimicrobial Combinations Against MDR Pathogens.**

Lanya K. Jalal ^1^, Laila I. Faqe Salih^1^, Payam B. Hassan^2*^

^1^Department of Medical Laboratory Sciences, College of Sciences, Charmo University, Sulaymaniyah 46001, Iraq

^2^Department of Biology, College of Science, University of Sulaimani, Sulaymaniyah, 46001, Kurdistan Region, Iraq

Author for correspondence: Laila Ibrahim Faqe Salih

Email: Laila.Ibrahim@Chu.edu.iq

**Table 1:** Biochemical characteristics (Gram reaction, oxidase, and catalase results) of *Enterococcus hiare*,*Morganella morganii,Kersteria gyiorum* and *klebsiella pneumoniae*.

| Bacterial species | Gram Type | Oxidase | Catalase |
| --- | --- | --- | --- |
| *M. morganii* | Gram-negative | Negative | Positive |
| *K. gyiorum* | Gram-negative | Negative | Positive |
| *E. hirae* | Gram-positive | Negative | Negative |
| *K. pneumoniae* | Gram-negative | Negative | Positive |
