# Supplementary material for: Enterococcus hirae‐Mediated ZnO and CuO/ZnO Nanoparticles: Synergistic Antimicrobial Combinations Against MDR Pathogens
Source: Int J Microbiol. 2026 Feb 1;2026:1969553. doi: 10.1155/ijm/1969553 (PMC12862103; doi:10.1155/ijm/1969553)
Supplement: Supplementary file 3 — Supporting Information 3 File S3 contains physicochemical characterization of synthesized nanoparticles, including Table S1: X‐ray diffraction (XRD) results summary, Table S2: Fourier transform infrared spectroscopy (FTIR) results summary, and Table S3: Energy‐dispersive X‐ray spectroscopy (EDS) results summary. [file IJM-2026-1969553-s003.docx]

**Supplementary File (S3)**

***Enterococcus hirae*-Mediated ZnO and CuO/ZnO Nanoparticles: Synergistic Antimicrobial Combinations Against MDR Pathogens.**

Lanya K. Jalal ^1^, Laila I. Faqe Salih^1^, Payam B. Hassan^2*^

^1^Department of Medical Laboratory Sciences, College of Sciences, Charmo University, Sulaymaniyah 46001, Iraq

^2^Department of Biology, College of Science, University of Sulaimani, Sulaymaniyah, 46001, Kurdistan Region, Iraq

Author for correspondence: Laila Ibrahim Faqe Salih

Email: Laila.Ibrahim@Chu.edu.iq

**Table 1: X**-ray Diffraction (XRD) Results Summary

| Sample | 2θ Peaks (°) | Miller Indices (hkl) | Reference ICDD |
| --- | --- | --- | --- |
| E‑ZnO NPs | 31.7, 34.4, 36.2, 47.5, 56.6, 62.8, 67.9, 69.1 | (010), (002), (011), (012), (110), (013), (112), (021) | 98‑011‑5755 |
| E‑ZnO/CuO NPs | Additional CuO peaks :33.0, 35.8, 53.8, 58.6) Overlapping peaks: 66.3, 68.2 | (102), (111), (202), (020)  (303), (112) | 98‑065‑3723 |

**Table 2:** Fourier Transform Infrared Spectroscopy (FTIR) Results Summary

| Peak Position (cm⁻¹) | Functional Group / Vibration | Interpretation |
| --- | --- | --- |
| 3432.65 | O–H stretching | Hydroxyl-containing biomolecules; reduction/capping |
| 2344.96 | Atmospheric CO₂ | Background FTIR signal |
| 1625.44 | C=C or –OH | Phenolics / polyphenols involvement |
| 1124.84, 1068.72 | C–O stretching | Alcohols, ethers, oxygenated biomolecules |
| 607.12, 462.65 | Zn–O | Formation of ZnO nanoparticles |
| 2922.27 | C–H stretching | Aliphatic chains; proteins/fatty acids |
| 1430.78, 1374.50 | Carboxylate (COO⁻) | Organic acids; biomolecule binding |
| 1632.89 | C=O or N–H bending | Protein/amine involvement |
| 593.34, 528.26 | Zn–O and Cu–O | Bimetallic oxide formation |

**Table 3:** Energy Dispersive X-ray Spectroscopy (EDS) Results summary.

| **Sample name** | **Elements Detected** | **Characteristic Peaks (keV)** |
| --- | --- | --- |
| E-ZnO NPs | Zinc (Zn) | 1.0, 8–9 |
|  | Oxygen (O) | 0–1 |
| E-CuO/ZnO NPs | Copper (Cu) | 0–1, 8–9 |
|  | Zinc (Zn) | 1, 8–9 |
